# Evaluation of NHS Practitioner Health: capturing mental health outcomes using five instruments

**DOI:** 10.1192/bjo.2021.926

**Published:** 2021-06-01

**Authors:** Kieran Simpson, Mark Ashworth, Sarah Roberts-Lewis, Salma Ayis

**Affiliations:** School of Population Health and Environmental Sciences, King's College London, UK

**Keywords:** Practitioner health, patient generated outcome measures, mental health change scores, psychometrics, standardised mental health outcome measures

## Abstract

**Background:**

NHS Practitioner Health is the England wide programme providing mental health and addiction healthcare to doctors and dentists. Outcomes are assessed using five measures.

**Aims:**

To contribute to a service evaluation of NHS Practitioner Health. To determine responsiveness to change and compare outcome measures.

**Method:**

Measures were completed at baseline and 6 months: Generalized Anxiety Disorder Assessment (GAD-7), Perceived Stress Scale (PSS), Patient Health Questionaire-9 (PHQ-9), Warwick-Edinburgh Mental Wellbeing scale (WEMWBS), Psychological Outcome Profiles (PSYCHLOPS). Responsiveness to change was determined using effect size with improvement threshold ≥0.80. Instruments were compared using Bland–Altman plots.

**Results:**

Our sample, *n* = 402; with 14 (3.5%) excluded for missing data; final sample, *n* = 388. All measures showed strong mean effect sizes: PSYCHLOPS 1.86 (95%CI 1.73–1.99), 75.8% ≥0.80; PSS 1.48 (1.34–1.62), 64.4% ≥0.80; WEMWBS 1.24 (1.13–1.35), 58.2% ≥0.80; GAD-7 1.07 (0.96–1.18), 52.8% ≥0.80; PHQ-9 0.86 (0.76–0.96), 52.8% ≥0.80. Findings were largely unchanged after stratification by diagnosis, presenting problem or therapy type. Fifty (12.9%) participants did not reach the threshold for improvement on any instrument. Bland–Altman plots indicated generally strong agreement between measures; combining PSYCHLOPS with WEMWBS maximised capture of improvement with only 3.6% of patients lying outside limits of agreement; GAD-7 was most likely to duplicate recovery scores of other measures.

**Conclusions:**

Patients attending the NHS Practitioner Health service demonstrated high levels of improvement in mental health scores. The patient-generated instrument produced higher change scores than standardised instruments. Combining PSYCHLOPS and WEMWBS captured 96% of patients with above threshold improvement; GAD-7 added little to overall recovery measurement.

NHS Practitioner Health is a national service providing care for doctors and dentists in England with mental health or addiction-related problems.^1^ Patients may self-refer into the service or be referred by their primary care or specialist doctors. The service is independent of any external agency, not part of the medical regulatory body and patients are offered the option of registering using a pseudonym as one of the features to ensure the strictest patient confidentiality.^1^ Individualised care consists of access to a broad range of expertise including talking therapy, professional advice and medication. The service uses five outcomes measures, four standardised and one patient generated. These are administered before the start of therapy and at other time points including at 6 months, although for many patients, treatment continues beyond 6 months.

Given the unique client group of healthcare professionals and a service with a primary aim to support practitioner patients in their return to work, we aimed to study the pattern of change at the 6-month stage of therapy as part of a NHS Practitioner Health service evaluation.

## Method

Our analysis was based on change scores obtained using five outcome measures.

### Design and setting

The five outcome measures consisted of four standardised measures: Generalised Anxiety Disorder Assessment (GAD-7),^2^ Perceived Stress Scale (PSS),^3^ Patient Health Questionaire-9 (PHQ-9),^4^ Warwick-Edinburgh Mental Wellbeing scale (WEMWBS)^5^; and one patient-generated measure: Psychological Outcome Profiles (PSYCHLOPS).^6^ Responsiveness to change was determined using effect size with the threshold for improvement set at ≥0.80.^7^ Instruments were compared using Bland–Altman plots.^8^ All data were routinely collected as part of service monitoring.

### Sample

All patients completing 6-month follow-up assessments at NHS Practitioner Health over a 15-month period, December 2017 to February 2019.

### Ethical approval

This work was part of an evaluation. All data were extracted from a confidential database and so were fully anonymised with no patient identifiers. All analysis was conducted in accordance with King's College London data security policies. As such, it fulfilled the Health Research Authority criteria for not requiring formal NHS ethical approval.

### Statistical methods

The effect size was calculated for each instrument by dividing the overall mean and 95% CI of the mean change scores at 6 months by the baseline standard deviation. Effect sizes for different instruments were compared.

The participants were then categorised in three ways. First, by clinical diagnosis made by the NHS Practitioner Health multidisciplinary team (according to diagnostic codes allocated following referral). Second, by categorising PSYCHLOPS Problem 1 free-text responses^6^ using thematic analysis. Thematic analysis was conducted by two independent reviewers (K.S. and another Masters student) who met to resolve any coding differences. If coding agreement could not be reached, a third reviewer (M.A.) was asked to agree a coding. Finally, the participants were categorised according to treatment modality. Effect sizes were calculated for each categorisation.

Bland–Altman plots were used to visually illustrate agreements between each pair of scales.^8^ Initially, the change in scores for each scale was standardised to *z*-scores using standard procedures to allow comparability. The *z*-score threshold for improvement was set at ≥0.80, and the percentage of individual *z*-scores above and below the threshold was calculated for each instrument. Paired *z*-score averages and differences were calculated between each pair of instruments and Bland–Altman plots were produced.

We then used Bland–Altman plots to identify the number of participants excluded by each pair combination of outcome measures. Based on five scales, this gives us ten pairs and 45 pair combinations for comparison. We identified participants who were excluded from the 95% limits of agreement by each pair of instruments. This enabled us to identify the pair combination that excluded the least number of participants, thus maximising the capture of participants above the improvement threshold. Conversely, we could identify which measure added the least information about improvement scores and may indicate redundancy.

The software SPSS (24.0) was used for calculation of effect sizes and Stata (16.0 BAPLOT module) was used for the production of Bland–Altman estimates and graphs.^9,10^

## Results

### Effect sizes

The sample consisted of 402 participants; 14 (3.5%) were excluded for missing data resulting in a final sample of 388. The mean age of participants was 41.0 years (s.d. = 9.5 years); 29% were men. The main clinical occupations of patients were general practitioners (72%) and hospital doctors (25%); 63% were in training grades.

**Fig. 1:**
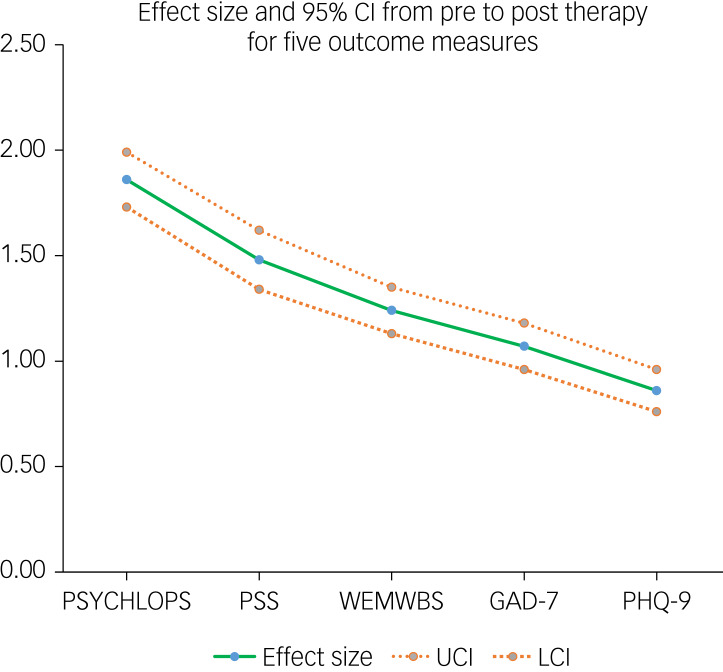
Change score effect size and 95% CI from pre- to post-therapy for five outcome measures. GAD-7, Generalized Anxiety Disorder Assessment; LCI, lower confidence interval; PHQ-9, Patient Health Questionaire-9; PSS, Perceived Stress Scale; PSYCHLOPS, Psychological Outcome Profiles; UCI, upper confidence interval; WEMWBS, Warwick-Edinburgh Mental Wellbeing scale.

All measures showed mean effect sizes ≥0.80 (see Fig. 1):

- PSYCHLOPS 1.86 (95% CI 1.73–1.99); 75.8% ≥0.80;
- PSS 1.48 (95% CI 1.34–1.62); 64.4% ≥0.80;
- WEMWBS 1.24 (95% CI 1.13–1.35); 58.2% ≥0.80;
- GAD-7 1.07 (95% CI 0.96–1.18); 52.8% ≥0.80;
- PHQ-9 0.86 (95% CI 0.76–0.96), 52.8% ≥0.80.

However, 50 (12.9%) participants did not reach the effect size threshold for improvement on any of the five instruments. PSYCHLOPS failed to detect above-threshold improvement in 24%.

PSYCHLOPS mean effect sizes for the main diagnostic categories were:

- Anxiety (*n* = 155): 1.93 (95% CI 1.72–2.15);
- Depression (*n* = 110): 1.91 (95% CI 1.67–2.15);
- Adjustment reaction (*n* = 61): 1.97 (95% CI 1.62–2.00);
- Dependency, drugs, alcohol (*n* = 19): 1.93 (95% CI 1.35–2.52).

PSYCHLOPS mean effect sizes for the main PSYCHLOPS (Problem 1) thematic categories were:

- Psychological difficulties (*n* = 326): 1.92 (95% CI 1.78–2.07);
- Adjustment reaction (*n* = 61): 1.97 (95% CI 1.62–2.00);
- Social difficulties (family, relationships, financial issues), (*n* = 29): 1.47 (95% CI 0.99–1.95);
- Work-related difficulties (*n* = 22): 1.98 (95% CI 1.43–2.53).

Note that patients could be allocated to more than one thematic category, resulting in a total larger than the total sample size.

PSYCHLOPS mean effect sizes for the main treatment modalities were:

- Cognitive–behavioural therapy (*n* = 234): 1.94 (95% CI 1.77–2.10);
- Case management (*n* = 121): 1.56 (95% CI 1.32–1.81);
- Psychotherapy (*n* = 30): 2.36 (95% CI 1.92–2.80).

The only non-overlapping effect sizes for PSYCHLOPS 95% CI applied to the relatively small sample of the psychotherapy treatment modality group, which had a larger effect size compared with the case management group.

Similarly, effect size 95% CI overlapped for each of the remaining four instruments categorised according to diagnostic category and thematic category (results not shown). Comparing psychotherapy with case management treatment modality for the remaining instruments, the 95% CI overlapped using PSS: 1.80 (95%CI 1.36–2.25) *v*. 1.23 (95%CI 0.98–1.48), respectively, and GAD-7: 1.29 (95% CI 0.91–1.67) *v*. 0.76 (95% CI 0.55–0.96), respectively. However, significantly larger effect sizes were found in the psychotherapy group compared with case management using WEMWBS: 1.68 (95% CI 1.28–2.08) *v*. 1.01 (95% CI 0.81–1.21) and PHQ-9: 1.25 (95% CI 0.91–1.59) *v*. 0.73 (95% CI 0.56–0.90).

### Bland–Altman plots

**Fig. 2:**
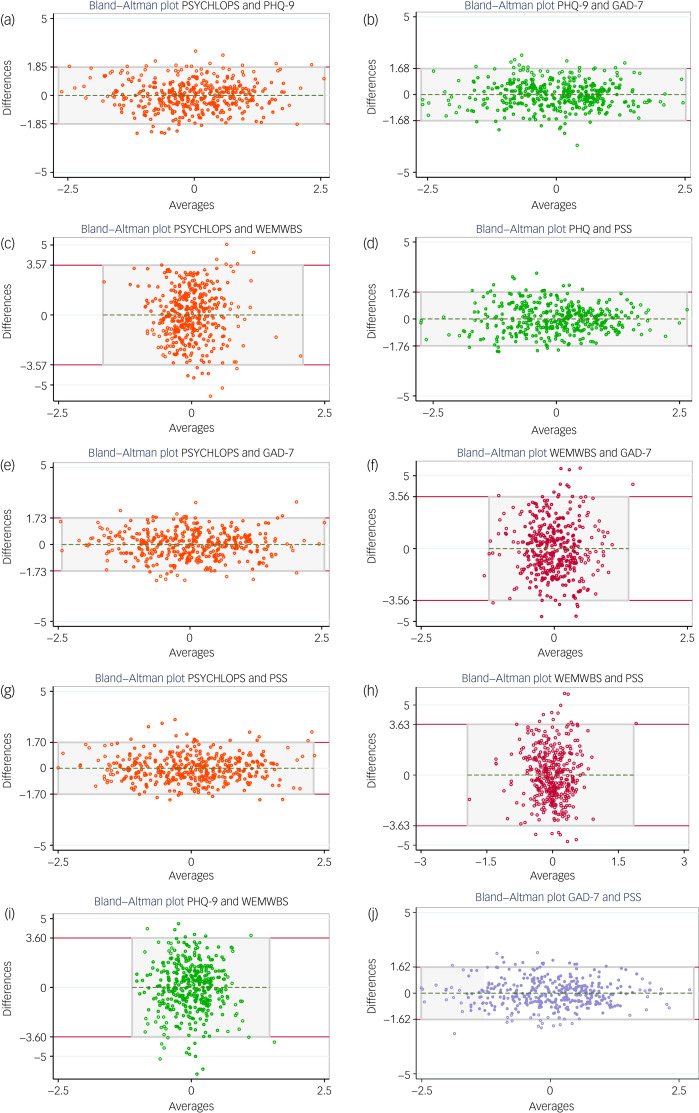
Bland–Altman plots displaying relationship between each pair of five outcome measures used to assess the response to therapy (each point represents a standardised change score). (a) Bland−Altman plot PSYCHLOPS and PHQ9; (b) Bland−Altman plot PHQ-9 and GAD-7; (c) Bland−Altman plot PSYCHLOPS and WEMWBS; (d) Bland−Altman plot PHQ and PSS; (e) Bland−Altman plot PSYCHLOPS and GAD-7; (f) Bland−Altman plot WEMWBS and GAD-7; (g) Bland−Altman plot PSYCHLOPS and PSS; (h) Bland−Altman plot WEMWBS and PSS; (i) Bland−Altman plot PHQ-9 and WEMWBS; and (j) Bland−Altman plot GAD-7 and PSS. GAD-7, Generalized Anxiety Disorder Assessment; PHQ-9, Patient Health Questionaire-9; PSS, Perceived Stress Scale; PSYCHLOPS, Psychological Outcome Profiles; WEMWBS, Warwick-Edinburgh Mental Wellbeing scale.

Bland–Altman plots that display the average of two measurements versus the difference between each pair are given in Fig. 2 for all ten pairs of the five scales. The scatter plots show on the y-axis the difference between the paired measurements, and on the *x*-axis the average of the two. The authors of the plots recommended that 95% of the data points should lie within the 95% limits of agreement (plus or minus 1.96, the s.d. of the mean difference between the two measurements) to indicate good agreement.^8^

**Fig. 3:**
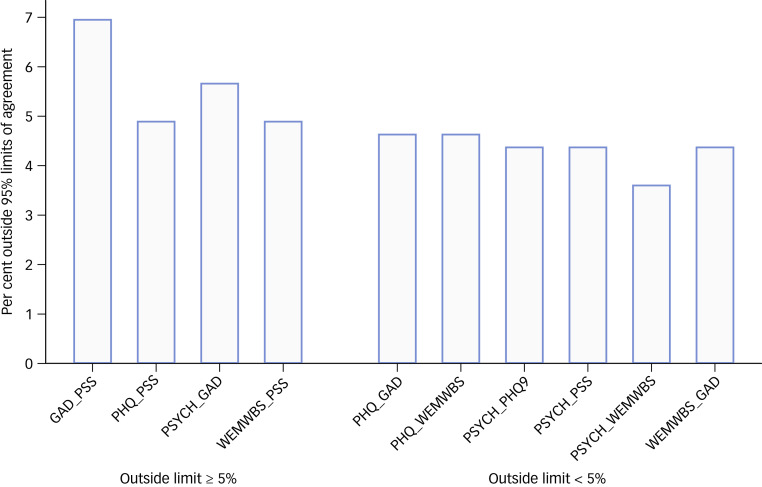
Bar charts presenting the percentage excluded by Bland–Altman 95% limits of agreement of each pair of the five instruments. GAD-7, Generalized Anxiety Disorder Assessment; PHQ-9, Patient Health Questionaire-9; PSS, Perceived Stress Scale; PSYCHLOPS, Psychological Outcome Profiles; WEMWBS, Warwick-Edinburgh Mental Wellbeing scale.

The proportions and numbers outside the 95% limits of agreements for each pair of scales together with the limits of agreement are reported in Table 1; Fig. 3 summarises the percentage of each measure excluded by Bland–Altman limits of agreement for each pair of instruments. The combination of GAD-7 and PSS resulted in 27 (7.0%) participant values outside the limits of agreement about significant improvement, the highest level of disagreement for all combinations (Table 1 and Fig. 3).

**Table 1:** Bland–Altman estimates for comparisons between pairs of instruments using standardised scores

Reference scale and comparison scale	Outside agreement limits, *n*	Outside agreement limits %	95% Limits of agreement	Averages lie between
PSYCHLOPS				
WEMWBS	14	3.61	(−3.57 to 3.57)	−1.65 and 2.10
PSS	17	4.38	(−1.70 to 1.70)	−2.51 and 2.32
PHQ-9	17	4.38	(−1.85 to 1.85)	−2.69 and 2.60
GAD-7	22	5.67	(−1.73 to 1.73)	−2.43 and 2.56
PHQ-9				
WEMWBS	18	4.64	(−3.60 to 3.60)	−1.11 and 1.47
GAD-7	18	4.64	(−1.68 to 1.68)	−2.63 and 2.52
PSS	19	4.90	(−1.76 to 1.76)	−2.76 and 2.67
WEMWBS				
GAD-7	17	4.38	(−3.56 to 3.56)	−1.22 and 1.39
PSS	19	4.90	(−3.63 to 3.63)	−1.94 and 1.85
PSS	27	6.90	(−1.62 to 1.62)	−2.55 and 2.79

GAD-7, Generalized Anxiety Disorder Assessment; PHQ-9, Patient Health Questionaire-9; PSS, Perceived Stress Scale; PSYCHLOPS, Psychological Outcome Profiles; WEMWBS, Warwick-Edinburgh Mental Wellbeing scale.

Combining PSYCHLOPS with WEMWBS maximised capture of improvement with only 3.6% of patients lying outside limits of agreement (Table 1 and Fig. 3).

**Fig. 4:**
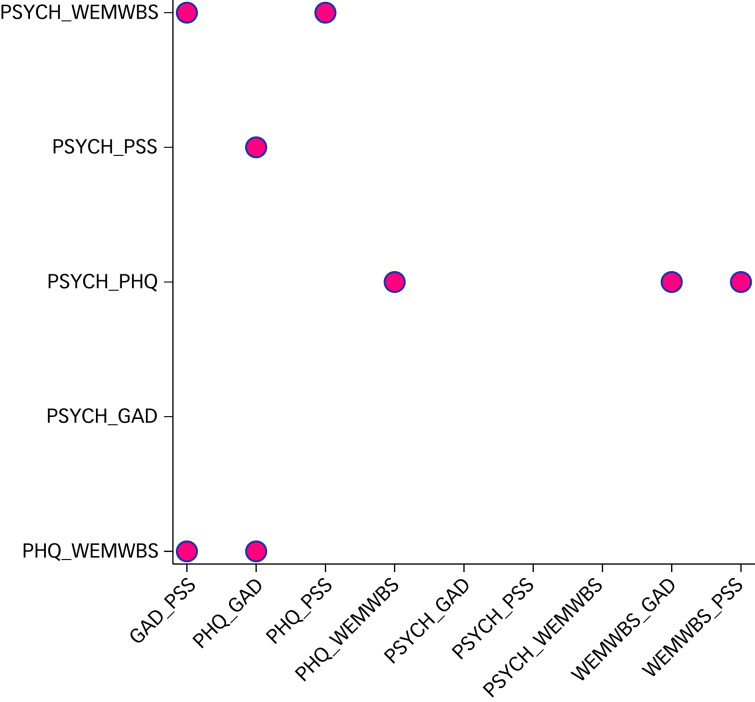
Scatter plots of two pairs of instruments that in combination have included all participants within the 95% Bland–Altman limits of agreement. Pairs of instruments are compared; pairings may include an instrument twice. GAD-7, Generalized Anxiety Disorder Assessment; PHQ-9, Patient Health Questionaire-9; PSS, Perceived Stress Scale; PSYCHLOPS, Psychological Outcome Profiles; WEMWBS, Warwick-Edinburgh Mental Wellbeing scale.

In further analysis of levels of agreement, we compared Bland–Altman plots for combinations of instrument pairs. There were 45 possible combinations of instrument pairs (four instruments). Eight of these combinations include all patients lying within the 95% limits of agreement (Fig. 4). In other words, patients lying outside the 95% limits of agreement for the reference instrument pair were included by a second instrument pair in eight combinations. Either PSYCHLOPS or WEMWBS appeared in all eight combinations. Further data on the combinations of instrument pairs is available in Supplementary File, Supplementary Table 1 available at https://doi.org/10.1192/bjo.2021.926.

## Discussion

### Main findings

The therapeutic intervention offered by NHS Practitioner Health has achieved relatively large change scores at the 6-month stage on all measures used as routine outcome assessment. Improvement on the patient-generated measure, PSYCHLOPS, was largest. Patient-generated measures are known to demonstrate greater responsiveness to change than standardised measures.^11^ The generally accepted reason for this feature of patient-generated measures is that by focusing on issues of personal concern, change is personalised and issues lacking personal significance do not act to dilute change scores.^11^ Although patient-generated and standardised measures generally show moderate convergent validity, standardised measures are required for setting diagnostic thresholds.

Categorisation of patients according to their primary clinical diagnosis, their self-described main problem in the free-text section of PSYCHLOPS or by treatment modality produced several patient subgroups. The largest diagnostic categories were ‘anxiety’ followed by ‘depression’, but there was no significant difference (based on overlapping CI) in PSYCHLOPS or other outcome measure effect sizes between any of the diagnostic categories.

The largest thematic category was ‘psychological difficulties’ with other categories such as ‘adjustment reaction’, ‘social difficulties’ or ‘work-related difficulties’ accounting for a much smaller proportion of patients. Again, there was no difference in PSYCHLOPS or other outcome measure effect sizes between these categories.

The main treatment modalities were cognitive–behavioural therapy or case management (a pragmatic mix of lead clinician support including advice, signposting, report writing, brokering employer meetings) for which there was no difference in outcome measure effect sizes. In the much smaller sample of patients receiving psychotherapy, three of the five instruments demonstrated a significantly greater effect size than for the case management group but overall differences were small. Taken together, these findings have not identified any subgroup of patients who perform notably better or notably worse following therapeutic intervention with the possible exception of patients receiving psychotherapy treatment.

Comparison of agreement between the five instruments was analysed using Bland–Altman plots. Overall, there was a high level of agreement based on pre-determined levels. Combinations of instruments increased the chances of capturing significant change. Our cautious interpretation is that the combination of PSYCHLOPS and WEMWBS had the highest capability for capturing significant change compared with other combinations. In the presence of one of these instruments, and any two others, all patients with significant change would be included. GAD-7, however, has shown the highest exclusion rate when paired with another instrument, and therefore may be a candidate for redundancy.

### Comparison with the literature

There are few international evaluations of physician health programmes based on comparable client groups or metrics. Some programmes confine their attention to the treatment of addiction whereas others have a broader remit.^12^ Given the difficulties of evaluating disparate services, many studies have focused on narrative reviews rather than quantitative comparison.^13^ Others have focused on specific outcomes such as return to work rates, reduction of suicide^14^ or abstinence rates for those with addiction-related problems.^15^ The context of physician health is also important with one systematic review noting high rates of depression among physicians compared with other branches of the profession^16^ although reported addiction rates were similar to those in the general population.^13^ Participants in physician health programmes are reported to be strongly motivated to recovery and this is borne out by high addiction recovery rates^12^ with dedicated physician programmes producing better results than standard addiction treatment offered to physicians.^17^

International programmes working with sick doctors have followed different models making it difficult to compare outcomes. Physician health programmes have been developed since the 1970s in the USA, mainly relating to managing doctors with addiction and engaging, assessing then referring patients for treatment in abstinence-based programmes, often residential and providing case management and adherence monitoring.^18^

In Spain, the Program for the Integral Care of the Ill Physician provides care specifically for doctors with psychiatric disorders or addictions, and appears to have been successful covering a broad range of specialities and ensuring supportive care avoiding regulatory body involvement where possible; however, in a 15-year review, there is no report of outcome measurement.^19,20^ In Ireland, the Practitioner Health Matters Programme found that the most frequent mental health problem among doctors seeking help was anxiety, followed by ‘burnout/stress’ and depression although outcome measures reporting is not provided.^21^ Other reports have included baseline psychometric testing to characterise physician patients at the point of referral but without outcome reporting.^22^ The European Association for Physician Health has collated a broad overview of studies evaluating physician health programmes but no others include outcome measurement.^23^

Few studies have reported on overall mental health recovery or improvement rates in physician health programmes.^24^ It is therefore difficult to provide a context to the improvement proportions (87% achieved the improvement threshold on at least one measure in our study) or size of the improvement (mean effect sizes up to 1.86 in our study, depending on the measure used). In a previous pilot evaluation of NHS Practitioner Health based on 150 participants, the effect sizes for the same five measures were smaller, ranging from 0.73 (PHQ-9) to 1.39 (PSYCHLOPS) although this was conducted during an earlier time period.^25^ The individualised nature of NHS Practitioner Health interventions may be best suited to individualised outcome measurement and make it difficult to draw international comparisons with other practitioner health programmes.

Studies in other settings such as therapy for common mental illness in primary care or clinical psychology services have reported effect sizes using outcome measures included in our study. For example, in a study of 114 patients attending a clinical psychology service in south London, the pre-, post-therapy effect size was 1.61 for PSYCHLOPS and 1.15 for the Hospital Anxiety and Depression Scale.^6^ Using the standardised response mean, WEMWBS, another measure showing high responsiveness to change in our study, was found to have similar change scores with a maximum of 1.35 (in a study of 85 patients in Perth and Kinross, Scotland).^26^

### Strengths and limitations of the present study

This evaluation reports on a large sample size compared with other reports of physician health programmes.^22^ However, the population is unique and likely to reflect distinctive features of UK healthcare regulatory bodies, working conditions and workplace support making the findings difficult to generalise for other national healthcare systems. Similarly, the individualised intervention is unique, guided by patient preference, which again may reduce generalisability of the findings.

The large sample in this study is based on patients completing psychometric outcome measures. Because of data access restrictions, we do not have demographic data on non-responders which may have introduced bias into our evaluation.

The presentation of outcomes based on five validated psychometric instruments and stratified according to clinical diagnostic categories and patient-generated thematic categories is unique. All change scores related to change at 6 months after starting therapy, although many patients continued treatment well beyond 6 months and we do not have data related to duration of therapy. Nor can we readily interpret the findings of significantly greater change (improvement) in patients treated with psychotherapy as opposed to case management because the sample of psychotherapy patients was much smaller (*n* = 30) and patient characteristics are likely to have differed substantially in each treatment modality. We therefore conclude that aggregated outcome scores do not provide sufficient information about the successful ‘ingredient’ of intervention. More detailed subgroup analysis was precluded as a condition for data access in order to avoid risk of identification during the course of service evaluation.

The majority of NHS Practitioner Health patients continue therapy beyond 6 months so our finding of lack of benefit for 50 patients on all five measures may be premature. We did not have access to data on return to work, time away from work or type of return (full time or part time), although work-related outcomes are likely to be of equal or greater importance to patients than outcome measure change scores.

### Implications

Mean reported change scores on all five instruments exceeded the pre-set threshold for change, indicating a moderate to strong effect of the intervention although our study is unable to determine which aspect of the broad range of therapies was most effective. Of the five instruments in use in the NHS Practitioner Health service, GAD-7 demonstrated the strongest duplication of other measures, implying that it may offer little additional benefit and may be a candidate for redundancy. However, it is clear that even the measure reporting the highest effect size failed to detect above-threshold improvement in 24%, a value which reduced to 13% when all five measures were included. Most services report non-responsiveness to talking therapy in about a third of patients suggesting that physician patients are less likely to be treatment resistant.^27,28^

Further study is needed to define the characteristics and alternative treatment options for participants not demonstrating recovery and to investigate delayed recovery trajectories.

## Data Availability

The data was obtained from a confidential and anonymised dataset which is not available to external researchers unless permission is granted by NHS Practitioner Health.

## Supplementary material

For supplementary material accompanying this paper visit http://dx.doi.org/10.1192/bjo.2021.926. click here to view supplementary material
