# Supplementary material for: Evaluation of NHS Practitioner Health: capturing mental health outcomes using five instruments
Source: BJPsych Open. 2021 Jun 1;7(4):e106. doi: 10.1192/bjo.2021.926 (PMC8220854; doi:10.1192/bjo.2021.926)
Supplement: Supplementary file 1 [file S2056472421009261sup001.docx]

**Supplementary File**

**Table 1 (supplementary)**

Data on the summary of levels of agreement presented in Figures 2-4 and Table 1 is provided in this Table. This table summarises the 45 possible combinations of instrument pairs.

| Comb. ID | Reference (Pair 1) | Outside Pair1, n | Outside Pair1, % | Pair2 | Outside Pair2, n | Outside Limits of Pair1 and Pair2, n | Comb. of 4 different=1* |
| --- | --- | --- | --- | --- | --- | --- | --- |
| 1 | PSYCH_WEMWBS | 14 | 3.61 | WEMWBS_PSS | 19 | 6 | 0 |
| 2 | PSYCH_WEMWBS | 14 | 3.61 | WEMWBS_GAD | 17 | 8 | 0 |
| 3 | PSYCH_WEMWBS | 14 | 3.61 | PSYCH_PSS | 17 | 2 | 0 |
| 4 | PSYCH_WEMWBS | 14 | 3.61 | PSYCH_GAD | 22 | 2 | 0 |
| 5 | PSYCH_WEMWBS | 14 | 3.61 | PHQ_WEMWBS | 18 | 6 | 0 |
| 6 | PSYCH_WEMWBS | 14 | 3.61 | PHQ_PSS | 19 | 0 | 1 |
| 7 | PSYCH_WEMWBS | 14 | 3.61 | PHQ_GAD | 18 | 1 | 1 |
| 8 | PSYCH_WEMWBS | 14 | 3.61 | GAD_PSS | 27 | 0 | 1 |
| 9 | PSYCH_PHQ | 17 | 4.38 | PSYCH_WEMWBS | 14 | 2 | 0 |
| 10 | PSYCH_PHQ | 17 | 4.38 | PSYCH_PSS | 17 | 2 | 0 |
| 11 | PSYCH_PHQ | 17 | 4.38 | PSYCH_GAD | 22 | 5 | 0 |
| 12 | PSYCH_PHQ | 17 | 4.38 | PHQ_WEMWBS | 18 | 0 | 0 |
| 13 | PSYCH_PHQ | 17 | 4.38 | PHQ_PSS | 19 | 3 | 0 |
| 14 | PSYCH_PHQ | 17 | 4.38 | PHQ_GAD | 18 | 3 | 0 |
| 15 | PSYCH_PHQ | 17 | 4.38 | WEMWBS_PSS | 19 | 0 | 1 |
| 16 | PSYCH_PHQ | 17 | 4.38 | WEMWBS_GAD | 17 | 0 | 1 |
| 17 | PSYCH_PHQ | 17 | 4.38 | GAD_PSS | 27 | 2 | 1 |
| 18 | PSYCH_PSS | 17 | 4.38 | WEMWBS_PSS | 19 | 4 | 0 |
| 19 | PSYCH_PSS | 17 | 4.38 | PHQ_PSS | 19 | 3 | 0 |
| 20 | PSYCH_PSS | 17 | 4.38 | GAD_PSS | 27 | 6 | 0 |
| 21 | PSYCH_PSS | 17 | 4.38 | WEMWBS_GAD | 17 | 1 | 1 |
| 22 | PSYCH_PSS | 17 | 4.38 | PHQ_WEMWBS | 18 | 1 | 1 |
| 23 | PSYCH_PSS | 17 | 4.38 | PHQ_GAD | 18 | 0 | 1 |
| 24 | WEMWBS_GAD | 17 | 4.38 | WEMWBS_PSS | 19 | 10 | 0 |
| 25 | WEMWBS_GAD | 17 | 4.38 | GAD_PSS | 27 | 1 | 0 |
| 26 | PHQ_GAD | 18 | 4.64 | WEMWBS_GAD | 17 | 1 | 0 |
| 27 | PHQ_GAD | 18 | 4.64 | PHQ_PSS | 19 | 4 | 0 |
| 28 | PHQ_GAD | 18 | 4.64 | GAD_PSS | 27 | 4 | 0 |
| 29 | PHQ_GAD | 18 | 4.64 | WEMWBS_PSS | 19 | 1 | 1 |
| 30 | PHQ_WEMWBS | 18 | 4.64 | WEMWBS_PSS | 19 | 9 | 0 |
| 31 | PHQ_WEMWBS | 18 | 4.64 | WEMWBS_GAD | 17 | 10 | 0 |
| 32 | PHQ_WEMWBS | 18 | 4.64 | PHQ_PSS | 19 | 2 | 0 |
| 33 | PHQ_WEMWBS | 18 | 4.64 | PHQ_GAD | 18 | 0 | 0 |
| 34 | PHQ_WEMWBS | 18 | 4.64 | GAD_PSS | 27 | 0 | 1 |
| 35 | PHQ_PSS | 19 | 4.90 | WEMWBS_PSS | 19 | 3 | 0 |
| 36 | PHQ_PSS | 19 | 4.90 | GAD_PSS | 27 | 7 | 0 |
| 37 | PHQ_PSS | 19 | 4.90 | WEMWBS_GAD | 17 | 1 | 1 |
| 38 | WEMWBS_PSS | 19 | 4.90 | GAD_PSS | 27 | 2 | 0 |
| 39 | PSYCH_GAD | 22 | 5.67 | WEMWBS_GAD | 17 | 1 | 0 |
| 40 | PSYCH_GAD | 22 | 5.67 | PSYCH_PSS | 17 | 7 | 0 |
| 41 | PSYCH_GAD | 22 | 5.67 | PHQ_GAD | 18 | 4 | 0 |
| 42 | PSYCH_GAD | 22 | 5.67 | GAD_PSS | 27 | 6 | 0 |
| 43 | PSYCH_GAD | 22 | 5.67 | WEMWBS_PSS | 19 | 2 | 1 |
| 44 | PSYCH_GAD | 22 | 5.67 | PHQ_WEMWBS | 18 | 1 | 1 |
| 45 | PSYCH_GAD | 22 | 5.67 | PHQ_PSS | 19 | 3 | 1 |

* there are 15 combinations (indicated by the value, 1) where the combination of instrument pairs consist of 4 different instruments; in 6 combinations, all participants were captured (‘Outside Limits of Pair1 and Pair2, n = 0).
